# Nutraceutical Approach for Preventing Obesity-Related Colorectal and Liver Carcinogenesis

**DOI:** 10.3390/ijms13010579

**Published:** 2012-01-05

**Authors:** Masahito Shimizu, Masaya Kubota, Takuji Tanaka, Hisataka Moriwaki

**Affiliations:** 1 Department of Medicine, Gifu University Graduate School of Medicine, Gifu 501-1194, Japan; E-Mails: samurai0201@yahoo.co.jp (M.K.); hmori@gifu-u.ac.jp (H.M.); 2 The Tohkai Cytopathology Institute: Cancer Research and Prevention (TCI-CaRP), Gifu 500-8285, Japan; E-Mail: takutt@toukaisaibou.co.jp

**Keywords:** obesity, colorectal cancer, hepatocellular carcinoma, chemoprevention, green tea catechins, branched-chain amino acids

## Abstract

Obesity and its related metabolic abnormalities, including insulin resistance, alterations in the insulin-like growth factor-1 (IGF-1)/IGF-1 receptor (IGF-1R) axis, and the state of chronic inflammation, increase the risk of colorectal cancer (CRC) and hepatocellular carcinoma (HCC). However, these findings also indicate that the metabolic disorders caused by obesity might be effective targets to prevent the development of CRC and HCC in obese individuals. Green tea catechins (GTCs) possess anticancer and chemopreventive properties against cancer in various organs, including the colorectum and liver. GTCs have also been known to exert anti-obesity, antidiabetic, and anti-inflammatory effects, indicating that GTCs might be useful for the prevention of obesity-associated colorectal and liver carcinogenesis. Further, branched-chain amino acids (BCAA), which improve protein malnutrition and prevent progressive hepatic failure in patients with chronic liver diseases, might be also effective for the suppression of obesity-related carcinogenesis because oral supplementation with BCAA reduces the risk of HCC in obese cirrhotic patients. BCAA shows these beneficial effects because they can improve insulin resistance. Here, we review the detailed relationship between metabolic abnormalities and the development of CRC and HCC. We also review evidence, especially that based on our basic and clinical research using GTCs and BCAA, which indicates that targeting metabolic abnormalities by either pharmaceutical or nutritional intervention may be an effective strategy to prevent the development of CRC and HCC in obese individuals.

## 1. Introduction

Obesity, which is the result of a positive energy balance, is a serious health problem throughout the world. The World Health Organization (WHO) estimates that currently, more than 1.5 billion adults worldwide are overweight, of which at least 500 million are obese [1]. Obesity is linked to several health disorders such as cardiovascular disease, hypertension, diabetes mellitus, and hyperlipidemia, which are collectively known as “metabolic syndrome”. In addition, mounting evidence indicates that obesity and its related metabolic abnormalities, especially diabetes mellitus, are associated with the development of certain types of human epithelial malignancies, including colorectal cancer (CRC) and hepatocellular carcinoma (HCC) [2–8]. On the basis of systematic reviews of epidemiological evidence as well as mechanistic interpretations and data from animal experimental models, the World Cancer Research Fund and American Institute for Cancer Research released a report in 2007 on the causal relationship between high body fatness and an increased risk of CRC [9]. A large-scale meta-analysis (221 datasets on 282,000 incidence cases) also revealed that the magnitude of risk for CRC was greater among obese men than non-obese men [10]. In a prospectively studied population of more than 900,000 American adults, the body mass index (BMI) was found to be significantly associated with higher rates of death from cancer, especially HCC, because the relative risk of death from HCC was significantly higher (4.52 times) among men with a BMI of at least 35.0 than those who had normal weight (95% confidence interval, 2.94–6.94) [11].

Several pathophysiological mechanisms that link obesity and colorectal and liver carcinogenesis have been shown, including the emergence of insulin resistance, alterations in the insulin-like growth factor-1 (IGF-1)/IGF-1 receptor (IGF-1R) axis, the state of chronic inflammation, induction of oxidative stress, and occurrence of adipocytokine imbalance [2–6]. On the other hand, these findings also suggest that targeting these pathophysiological disorders via nutritional or pharmaceutical intervention might be an effective and promising strategy to inhibit obesity-related carcinogenesis. For instance, a 3-hydroxy-3-methylglutaryl coenzyme A (HMG-CoA) reductase inhibitor pitavastatin, which is widely used to treat hyperlipidemia, prevents obesity-related colorectal and liver carcinogenesis by attenuating chronic inflammation [12,13]. Captopril and telmisartan, which are anti-hypertensive drugs, also suppress the development of colonic preneoplastic lesions in obese and diabetic mice, and this suppression is associated with the reduction of oxidative stress and chronic inflammation [14].

In recent years, green tea catechins (GTCs) have received considerable attention because of their beneficial effects: they improve metabolic abnormalities and prevent cancer development [15–19]. Dietary supplementation with branched-chain amino acids (BCAA; leucine, isoleucine, and valine), which can prevent progressive hepatic failure in patients with chronic liver disease by improving insulin resistance [20–22], also reduces the risk of HCC in such patients who are obese [8]. In this article, we review the many mechanisms by which obesity and the related metabolic abnormalities influence the development of CRC and HCC while especially focusing on the emergence of insulin resistance and the subsequent inflammatory cascade. We also prove that the nutraceutical approach using GTCs and BCAA might be effective in preventing obesity-related carcinogenesis in both the colorectum and liver.

## 2. Potential Pathophysiological Mechanisms Linking Obesity and the Development of CRC

Obesity is the main determinant of insulin resistance and hyperinsulinemia, which is a risk factor for CRC [23]. Insulin itself and the signal transduction network it regulates have important roles in oncogenesis [24,25]. In animal models, insulin stimulates the growth of CRC cells while also promoting CRC tumor growth [26,27]. In addition, insulin resistance increases the biological activity of IGF-1, an important endocrine and paracrine regulator of tissue growth and metabolism. The binding of insulin and IGF-1 to the cell-surface receptors, insulin receptor and IGF-1R, respectively, on tumors and precancerous cells activates the phosphatidylinositol 3-kinase (PI3K)/Akt pathway, which is responsible for cellular processes like growth, proliferation, and survival [24,25]. Alterations in the IGF/IGF-1R axis caused by insulin resistance contribute to the development of CRC [28]. IGF-1 is positively correlated with body fat and waist circumference [29]. Moreover, insulin resistance and increased adipose mass create an oxidative environment in the tissues that upregulates the expression of various pro-inflammatory cytokines, including tumor necrosis factor-α (TNF-α) and interleukin-6 (IL-6), which stimulate tumor growth and progression [30–34]. Increased oxidative stress promotes damage to cell structures, including DNA, and activates the PI3K/Akt pathway, and both these processes play a key role in cancer development [35,36]. Therefore, insulin resistance and the subsequent inflammatory cascade involving increased oxidative stress are regarded as important factors in the development of obesity-associated CRC.

**Figure 1:**
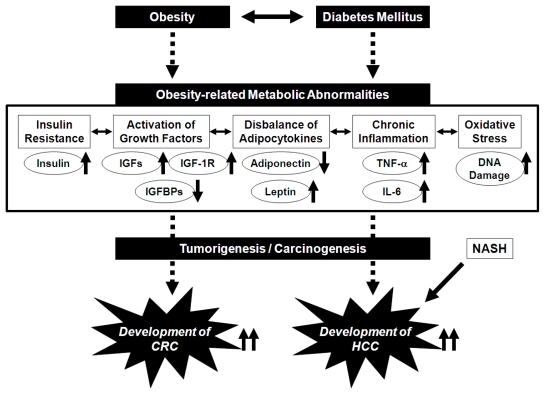
Proposed mechanisms linking obesity and its related metabolic abnormalities to the development of colorectal cancer (CRC) and hepatocellular carcinoma (HCC).

Excess production of storage lipids causes an adipocytokine imbalance, which entails increased levels of leptin and decreased levels of adiponectin in the serum, for example. This imbalance may also be related to obesity-associated carcinogenesis [37,38]. Leptin stimulates cell growth in CRC [39]. An epidemiologic study by Stattin *et al*. [40] suggested an association between circulating leptin levels and the development of CRC. TNF-α and IL-6 increase the levels of leptin, while leptin influences inflammatory responses, possibly by triggering the release of TNF-α and IL-6 [41–43]. These findings suggest that the pathophysiological abnormalities caused by obesity cooperatively aggravate the risk of cancers, including CRC, in obese individuals (Figure 1).

## 3. Potential Pathophysiological Mechanisms Linking Obesity, Non-Alcoholic Fatty Liver Disease/Non-Alcoholic Steatohepatitis, and the Development of HCC

Several pathophysiological mechanisms linking obesity, steatosis, and liver carcinogenesis have been shown, including insulin resistance and the subsequent inflammatory cascade. Insulin induces HCC cells to proliferate and resist apoptosis [44,45]. Insulin resistance raises the risk for recurrence of HCC after curative radiofrequency ablation in hepatitis C virus-positive patients [46]. Insulin resistance also leads to an increased expression of TNF-α and its dysregulation is associated with the development of steatosis and inflammation within the liver [47]. Activation of the IGF/IGF-1R axis is involved with liver carcinogenesis [48,49]. High levels of serum leptin, which stimulates the growth of HCC cells [50], increase the risk of HCC recurrence after curative treatment [51]. These findings suggest that in addition to colorectal carcinogenesis, obesity and its related metabolic abnormalities also play an important role in the development of HCC (Figure 1).

Non-alcoholic fatty liver disease (NAFLD), which is known to be a hepatic manifestation of metabolic syndrome, is the most common form of chronic liver disease in developed countries [52,53]. It covers a spectrum of disorders ranging from simple steatosis to non-alcoholic steatohepatitis (NASH), which can progress to cirrhosis and thus HCC (Figure 1) [52,53]. Retrospective data suggest that in as many as 4–27% of cases, NASH progresses to HCC after cirrhosis develops [53,54]. Insulin resistance is considered a critical factor in the etiology of NASH [55]. The flux of free fatty acids to the liver and insulin resistance lead to hepatic fat accumulation, which causes inflammatory changes in the liver [56,57]. Enhanced TNF-α expression and increased leptin levels are also found in patients with NASH [58,59]. In addition, Wong *et al*. [60] recently reported interesting results from a cross-sectional study, indicating that NASH is associated with a high prevalence of colorectal adenomas and advanced neoplasms. This finding may suggest that in addition to HCC, NASH may be associated with an increased risk of CRC.

## 4. Preventive Effects of GTCs on the Metabolic Abnormalities and Cancer Development

Numerous studies have indicated that tea catechins, especially GTCs, are beneficial for various reasons, such as their anti-obesity effects [15]. A recent meta-analysis of clinical trials reported that GTCs help reduce body weight [61]. The underlying mechanisms include an increase in energy expenditure, stimulation of fatty acid oxidation, and reduction of nutrient absorption [62]. The effects of GTCs whereby they suppress metabolic syndrome have also been investigated in laboratory, epidemiological, and intervention studies [63,64]. In a rodent model of obesity and diabetes, treatment with green tea or its constituents was found to result in significantly reduced body weight and, therefore, improved hyperglycemia, hyperinsulinemia, hyperleptinemia, hepatic steatosis, and liver dysfunction [65–67]. GTCs supplementation was also found to decrease plasma levels of insulin, TNF-α, and IL-6 in a rat insulin resistance model [68]. These reports suggest that long-term treatment with GTCs may be effective for preventing the progression of obesity-related metabolic disorders.

In addition to the anti-obesity effects, GTCs possess anti-cancer and cancer-preventive properties [16–19]. Intervention studies provide clear evidence of the chemopreventive effects of tea preparations [69,70]. A pilot study also showed that GTCs successfully prevent colorectal adenomas, the precancerous lesions of CRC, after polypectomy [71]. Several properties of GTCs are responsible for their anti-cancer and cancer-preventive effects, including their antioxidant and anti-inflammatory properties [16,72]. An increasing number of studies have reported that GTCs, especially the major biologically active component in green tea (−)-epigallocatechin gallate (EGCG), inhibit proliferation of and induce apoptosis among cancer cells by modulating the activities of different receptor tyrosine kinases (RTKs) and their downstream signaling pathways, including the Ras/extracellular signal-regulated kinase (ERK) and PI3K/Akt signaling pathways [17–19,73,74]. EGCG suppresses cell growth by inhibiting the activation of IGF-1R, a member of the RTK family, in human CRC and HCC cells, and this inhibition is associated with a decrease in the expression of IGF-1/2, but an increase in the expression of IGF-binding protein-3 (IGFBP-3), which negatively controls the function of the IGF/IGF-1R axis [49,75]. EGCG also prevents carbon tetrachloride-induced hepatic fibrosis in rats by inhibiting IGF-1R expression [76]. These reports indicate that the IGF/IGF-1R axis, which plays a critical role in both cancer development and obesity-induced pathological events [24,25], might be a critical target of GTCs.

## 5. Preventive Effects of BCAA on Metabolic Abnormalities and HCC in Obese, Cirrhotic Patients: Results Form the LOTUS Study

Because the liver, an important target organ of insulin, plays a critical role in regulating metabolism, patients with chronic liver diseases often suffer from several nutritional and metabolic disorders, such as protein-energy malnutrition and insulin resistance [77–80]. Decreased serum levels of BCAA and albumin are associated with a high incidence of liver cirrhosis, while supplementation with BCAA has been shown to improve protein malnutrition and increase the serum albumin concentration in cirrhotic patients [20,77,78]. In addition, recent experimental studies have revealed that BCAA improves insulin resistance and glucose tolerance [81–83]. She *et al*. [81] reported that mitochondrial branched-chain aminotransferase knock out mice, which show a significant elevation in the serum BCAA level, exhibit decreased adiposity and remarkable improvements in glucose and insulin tolerance. BCAA has favorable effects on glucose metabolism not just in the liver but also in skeletal muscle and adipose tissue [84–86]. In the liver, BCAA activates liver-type glucokinase and glucose transporter (GLUT)-2, while suppressing the expression of glucose-6-phosphatase, which catalyzes the final steps of gluconeogenesis [84]. On the other hand, BCAA promotes glucose uptake through activation of PI3K and subsequent translocation of GLUT1 and GLUT4 to the plasma membrane in the skeletal muscle [86]. Moreover, in mice fed a high-fat diet, BCAA supplementation ameliorated insulin resistance by improving adipocytokine imbalance, inhibiting lipid accumulation in the liver, and increasing the hepatic levels of peroxisome proliferator-activated receptor-α [87,88]. Several clinical trials have also reported that oral BCAA supplementation improves glucose tolerance and insulin resistance in patients with chronic liver disease [22,89,90].

The Long-Term Survival Study (LOTUS) was a large-scale (*n* = 622) multicenter randomized controlled trial conducted from 1997 to 2003 in Japan to investigate the effects of supplemental BCAA therapy on event-free survival in patients with decompensated cirrhosis. In this trial, oral supplementation with a BCAA preparation was found to significantly prevent progressive hepatic failure and improve event-free survival [20]. Moreover, subset analysis from this trial demonstrated that long-term oral supplementation with BCAA is associated with a reduced frequency of HCC in obese patients (BMI score ≥ 25, *P* = 0.008) with decompensated cirrhosis [8]. What could the mechanisms of action of BCAA in the prevention of HCC have been? It seems reasonable to consider that the improvement of glucose metabolism by BCAA contributes to a decrease in the HCC incidence in obese cirrhotic patients because these patients generally have a particularly high incidence of hyperinsulinemia and insulin resistance [79,80]. In addition, Hagiwara *et al*. [91] recently reported significant findings that BCAA suppresses insulin-induced proliferation of HCC cells by inhibiting the insulin-induced activation of the PI3K/Akt pathway and the subsequent anti-apoptotic pathway. The precise mechanisms of action of BCAA in relation to carcinogenesis are explained in detail in the following sections.

## 6. Prevention of Obesity-Related CRC via the Nutraceutical Approach—GTCs and BCAA Effectively Prevent Obesity-Related Colorectal Carcinogenesis

Recent evidence indicates that increased body fatness and BMI are associated with an increased risk of CRC [4,5,9–11]. In contrast, studies have provided convincing evidence that dietary habits, especially high fruit and vegetable consumption, may reduce the risk of this malignancy [92]. Hirose *et al.* [93] established a useful preclinical model to determine the underlying mechanisms of how specific agents prevent the development of obesity-related CRC. The model used was C57BL/KsJ-*db/db* (*db/db*) mice, which are a genetically altered animal model with phenotypes of obesity and diabetes mellitus [94]. These mice have hyperlipidemia, hyperinsulinemia, and hyperleptinemia and are susceptible to the colonic carcinogen azoxymethane (AOM) because AOM-induced colonic precancerous lesions, aberrant crypt foci (ACF) and β-catenin accumulated crypts (BCAC), develop to a significantly greater extent in these mice than in the genetic control mice [93]. The colonic mucosa of *db/db* mice expresses high levels of IGF-1R, the phosphorylated (activated) form of IGF-1R (*p*-IGF-1R), β-catenin, and cyclooxygenase-2 (COX-2) [95]. Dietary supplementation with certain types of flavonoids, such as citrus compounds, suppresses the development of these putative lesions for CRC in the *db/db* mice [96–98].

**Figure 2:**
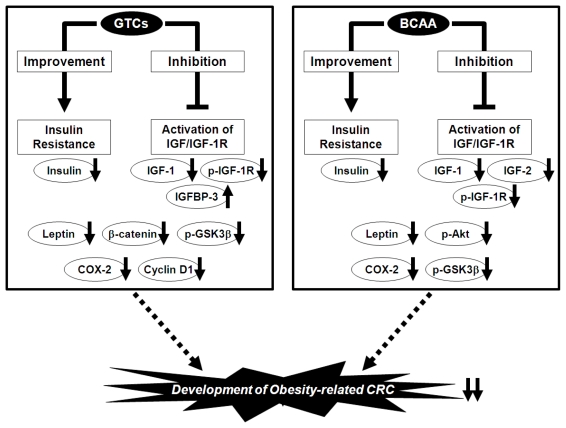
Mechanisms of action of green tea catechins (GTCs) and branched-chain amino acids (BCAA) in the inhibition of obesity-related colorectal carcinogenesis.

We used this experimental model to investigate in detail the effects of EGCG and BCAA on the prevention of obesity-related colorectal carcinogenesis. We found that drinking water with EGCG significantly decreased the number of ACF and BCAC, which accumulate the IGF-1R protein, and this decrease was associated with inhibited expression of IGF-1R, *p*-IGF-1R, the phosphorylated form of glycogen synthase kinase-3β (GSK-3β), β-catenin, COX-2, and cyclin D1 on the colonic mucosa [95]. EGCG also increased the serum level of IGFBP-3 while decreasing the serum levels of IGF-1, insulin, triglycerides, total cholesterol, and leptin [95]. In accordance with this study, supplementation with BCAA also caused a significant reduction in the number of ACF and BCAC compared with the control diet-fed groups by inhibiting the phosphorylation of IGF-1R, GSK-3β, and Akt on the colonic mucosa [99]. The serum levels of insulin, IGF-1, IGF-2, triglycerides, total cholesterol, and leptin were also decreased [99]. These findings suggest that both EGCG and BCAA effectively suppress the development of premalignant CRC lesions by suppressing the IGF/IGF-1R axis; improving hyperlipidemia, hyperinsulinemia, and hyperleptinemia; and inhibiting the expression of COX-2, which is involved in CRC development because it mediates inflammatory signaling pathways and can therefore be an important target for chemoprevention (Figure 2) [100].

## 7. Prevention of Obesity-Related HCC via the Nutraceutical Approach—BCAA and GTCs Effectively Prevent Obesity-Related Liver Carcinogenesis

In addition to established risk factors such as hepatitis and alcohol consumption, obesity and its related metabolic abnormalities increase the risk of HCC [6–8,11]. NASH is also an important pathological condition when considering the prevention of obesity-related HCC because it progresses to cirrhosis and finally develops into HCC [53,54]. In order to elucidate the pathogenesis of obesity-and NASH-associated HCC and evaluate the mechanisms of how chemopreventive agents suppress these diseases, we developed a useful preclinical model using *db/db* mice and a liver carcinogen *N*-diethylnitrosamine (DEN) [101]. We found that *db/db* mice, which have severe steatosis, are more susceptible to DEN-induced liver tumorigenesis than the genetic control mice, and this oncogenic sensitivity is associated with the activation of the IGF/IGF-1R axis and induction of chronic inflammation in the liver [13,101–103].

**Figure 3:**
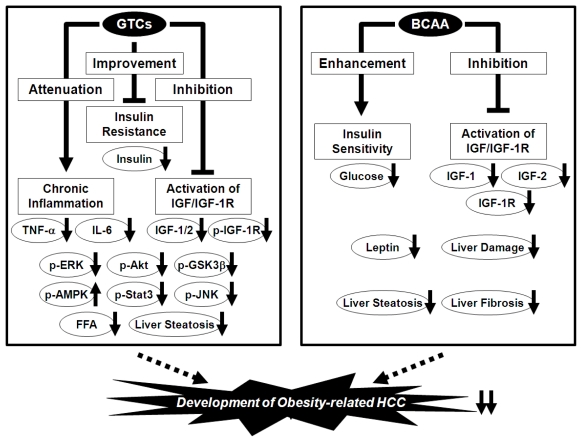
Mechanisms of action of GTCs and BCAA in the inhibition of obesity-related liver carcinogenesis.

Using this experimental model, we also investigated the possible inhibitory effects of BCAA and EGCG on obesity-related liver tumorigenesis. We found that BCAA supplementation significantly suppressed the development of hepatic preneoplastic lesions, known as foci of cellular alteration (FCA), in obese and diabetic *db/db* mice by inhibiting the expression of IGF-1, IGF-2, and IGF-1R in the liver [101]. The development of liver neoplasms, including hepatic adenoma and HCC, was also reduced by BCAA supplementation and this was associated with improvement of insulin resistance, reduction of serum levels of leptin, and attenuation of hepatic steatosis and fibrosis [101]. Yoshiji *et al*. [104] also reported that the chemopreventive effect exerted by BCAA supplementation against HCC in obese and diabetic rats was associated with the suppression of vascular endothelial growth factor expression and hepatic neovascularization. In addition, drinking water containing EGCG significantly inhibited the development of FCA and hepatic adenoma, and improved hepatic steatosis [103]. The serum levels of insulin, IGF-1, and IGF-2 and the phosphorylation of the IGF-1R, ERK, Akt, and GSK-3β proteins in the liver were reduced by EGCG consumption [103]. EGCG also decreased the levels of free fatty acids and TNF-α in the serum and the expression of TNF-α, IL-6, IL-1β, and IL-18 mRNAs in the liver, indicating that it prevents obesity-related liver tumorigenesis by inhibiting the IGF/IGF-1R axis, improving hyperinsulinemia, and attenuating chronic inflammation [103]. Thus, both BCAA and GTCs may be useful for the chemoprevention of liver carcinogenesis in obese individuals (Figure 3).

## 8. Conclusions

In the present social and medical circumstances, the consequences of obesity and its related metabolic abnormalities, including cancer, are critical issues that need to be resolved. Among human cancers, CRC and HCC are the most representative malignancies affected by obesity. In this review, we indicate the possibility that the nutraceutical approach for targeting and restoring metabolic homeostasis may be a promising strategy to prevent the development of obesity-related CRC and HCC. Tea catechins, especially GTCs, are considered one of the most practical agents for the prevention of obesity-related carcinogenesis because the safety and efficacy of GTCs as chemopreventive agents have been demonstrated in recent interventional trials [69,71]. BCAA is also a feasible agent because its preparations are widely used in clinical practice for patients with chronic liver diseases, and a randomized controlled trial has shown that BCAA supplementation can prevent HCC in such patients who are obese [8,20]. Thus, active intervention using GTCs and BCAA might be an effective approach for the chemoprevention of obesity-related CRC and HCC.
